# miR-27 Negatively Regulates Pluripotency-Associated Genes in Human Embryonal Carcinoma Cells

**DOI:** 10.1371/journal.pone.0111637

**Published:** 2014-11-04

**Authors:** Heiko Fuchs, Matthias Theuser, Wasco Wruck, James Adjaye

**Affiliations:** 1 Institute for Stem Cell Research and Regenerative Medicine, Faculty of Medicine, Heinrich Heine University, Duesseldorf, Germany; 2 Department of Vertebrate Genomics, Molecular Embryology and Aging Group, Max Planck Institute for Molecular Genetics, Berlin, Germany; National Cancer Institute, United States of America

## Abstract

Human embryonic stem cells and human embryonal carcinoma cells have been studied extensively with respect to the transcription factors (OCT4, SOX2 and NANOG), epigenetic modulators and associated signalling pathways that either promote self-renewal or induce differentiation in these cells. The ACTIVIN/NODAL axis (SMAD2/3) of the TGFß signalling pathway coupled with FGF signalling maintains self-renewal in these cells, whilst the BMP (SMAD1,5,8) axis promotes differentiation. Here we show that miR-27, a somatic-enriched miRNA, is activated upon RNAi-mediated suppression of OCT4 function in human embryonic stem cells. We further demonstrate that miR-27 negatively regulates the expression of the pluripotency-associated ACTIVIN/NODAL axis (SMAD2/3) of the TGFß signalling pathway by targeting *ACVR2A*, *TGFßR1* and *SMAD2*. Additionally, we have identified a number of pluripotency-associated genes such as *NANOG*, *LIN28*, *POLR3G* and *NR5A2* as novel miR-27 targets. Transcriptome analysis revealed that miR-27 over-expression in human embryonal carcinoma cells leads indeed to a significant up-regulation of genes involved in developmental pathways such as TGFß- and WNT-signalling.

## Introduction

Human embryonic stem cells (hESC), derived from the inner cell mass of blastocysts, have the potency to self-renew and differentiate into cells representative of all three germ layers [1]. A network of core transcription factors (TFs) including OCT4, NANOG, KLF4, LIN28 and SOX2 promote the undifferentiated state of both hESC and human embryonal carcinoma cells (hEC) via inducing and sustaining expression of stem cell related genes and simultaneously suppressing expression of somatic enriched genes. hEC are malignant tumor cells derived from teratocarcinomas and are considered the malignant counterparts of hESC. Both, hEC and hESC show a high degree of overlap in their transcriptomes. We previously demonstrated that FGF2 promotes autocrine signalling of TGFß receptor (TGFßR) ligands, such as *INHBA* and *TGFß1* inboth cell types [2], [3]. It has been shown that ACTIVIN A, one of the factors secreted by mouse embryonic fibroblasts (MEFs), is necessary for maintaining self-renewal and pluripotency in hESC [2], [4]. ACTIVIN A is a homodimer consisting of two subunits of INHIBIN beta A (*INHBA*). Like TGFß1 and NODAL, INHBA activates the SMAD2/3 branch of the TGFß signalling pathway which in turn activates pluripotency associated genes such as *NANOG* [2], [5], [6]. During differentiation, activated BMP4, binds its receptor, resulting in the activation of the SMAD1/5/8 branch of the TGFß-signalling pathway and hence expression of somatic enriched genes.

To date, additional genes have been reported to support self-renewal in hESC. The orphan nuclear receptor *NR5A2* (also known as liver receptor homolog *LRH-1*) activates *OCT4* expression in embryonic stem cells and human embryonal carcinoma cells [7], [8]. Remarkably, *OCT4* can be substituted by *NR5A2* when generating induced pluripotent stem (iPS) cells [9], [10]. Down-regulation of the RNA polymerase III subunit, *POLR3G*, a downstream target gene of OCT4 and NANOG, promotes the differentiation of hESC and iiPS [11].

Pluripotency can be induced in somatic cells by the ectopic expression of either *OCT4*, *SOX2*, *KLF4* and *MYC* (OSKM) or *OCT4*, *SOX2*, *LIN28* and *NANOG* (OSLN) [12], [13]. This implies that *KLF4* and *MYC* can be substituted by *LIN28* and *NANOG*. Unlike NANOG, the RNA-binding protein LIN28 operates at the post-transcriptional level. LIN28 interacts with polyribosomes and promotes the translation of mRNAs, such as *OCT4*, by regulating its stability [14], [15]. Moreover, LIN28 has been recently reported to establish the pluripotent state by binding the precursor form of the microRNA let-7 (pre-let-7), a well-studied microRNA that promotes differentiation. This binding event prevents maturation of let-7 due to uridylation and simultaneous degradation [16], [17].

microRNAs (miRNAs) are short (20–24 nt) single-stranded endogenously expressed RNAs that inhibit the translation of messenger RNAs predominantly due to imperfect binding to the 3′-UTR of their target genes [18]. In general, a single miRNA has the capacity to suppress hundreds of mRNA targets. miRNAs play an important role in regulating developmental and physiological processes, lineage as well as stem cell commitment. A number of miRNAs highly expressed in both ESCs and iPS have been identified in vertebrates [19], [20]. In human, OCT4, SOX2 and NANOG promote the expression of stem-cell enriched miRNAs, for example, the polycistronic miR-302/367 cluster [21]. miR-302 inhibits the translation of mRNAs inducing differentiation, including *NR2F2* (an antagonist of OCT4), *ZEB1* (Inhibitor of E-CADHERIN)and *BMPR2* (inducing SMAD1/5/8 signalling) [22]–[24]. BMP4 is a negative regulator of miR-302/367 [23]. Not surprisingly, a higher reprogramming efficiency has been achieved using a combination of *OCT4*, *SOX2*, *KLF4* and *MYC* together with miR-302/367 [25].

In contrast, a number of somatic miRNAs have been reported to act like an off-switch of self-renewal. For example, miRNA let-7 down-regulates LIN28, MYC, CDK1 and HMGA2 [16], [26], [27]. Beside let-7, the tumor suppressor oncogene TP53 activates miR-145 which in turn inhibits translation of *OCT4, SOX2, KLF4* and *LIN28* [28]. Another miRNA regulated by TP53, miR-34, has been reported to target SOX2 and NANOG [29].

In this study, we focus on the somatic-enriched microRNA, miR-27. In vertebrates two paralogs of miR-27, i.e. miR-27a and miR-27b, which only differ by one nucleotide, have been described. Both paralogs are transcribed within a polycystronic cluster together with miR-23 and miR-24. miRNA profiling revealed that the expression level of miR-27 increases in hESC undergoing endoderm priming and hepatocyte differentiation [30]. Other studies have shown that miR-27 is up-regulated during osteoblast differentiation and that miR-27 is highly expressed in endothelial cells [31], [32]. To date, an increasing number of functions of miR-27 have been reported. miR-27 prevents adipogenic differentiation by targeting two main regulators of adipogenesis, the peroxisome proliferator-activated receptor gamma (PPARγ) and C/EBP alpha [33]. miR-27 promotes myogenic differentiation by silencing PAX3 in muscle progenitor cells [34]. RUNX1, an inhibitor of granulocyte differentiation, has been confirmed as a miR-27 target [35]. miR-27 expression has also been linked to cancer, it inhibits the tumor suppressor FOXO1 in endometrial cancer [36]. miR-27 activates metastasis in human gastric cancer cells by activating the expression of *ZEB1*, *ZEB2* and *VIM* thus leading to an induction of epithelial-to-mesenchymal transition [37].

Here, we report a novel role of miR-27 as a negative modulator of self-renewal and pluripotency. using the hESC line, H1 and the hEC line, NCCIT as cellular models.

Employing an EGFP-based sensor approach, we show that miR-27 targets three genes of the ACTIVIN/TGFß branch of TGFß signalling pathway, namely: ACVR2, TGFßR1 and their downstream target SMAD2. Moreover, we demonstrate that *LIN28* and *NANOG* as well as *POLR3G* and *NR5A2* are target genes of miR-27. Transient over-expression of miR-27 in hEC, led to decreased levels of OCT4 mRNA and protein. Furthermore, siRNA-mediated ablation of OCT4 function in the hESC line H1 led to the activation of miR-27 expression and loss of self-renewal and pluripotency.

## Results

### ACVR2A, TGFßR1 and SMAD2 are direct targets of miR-27

**Figure 1:**
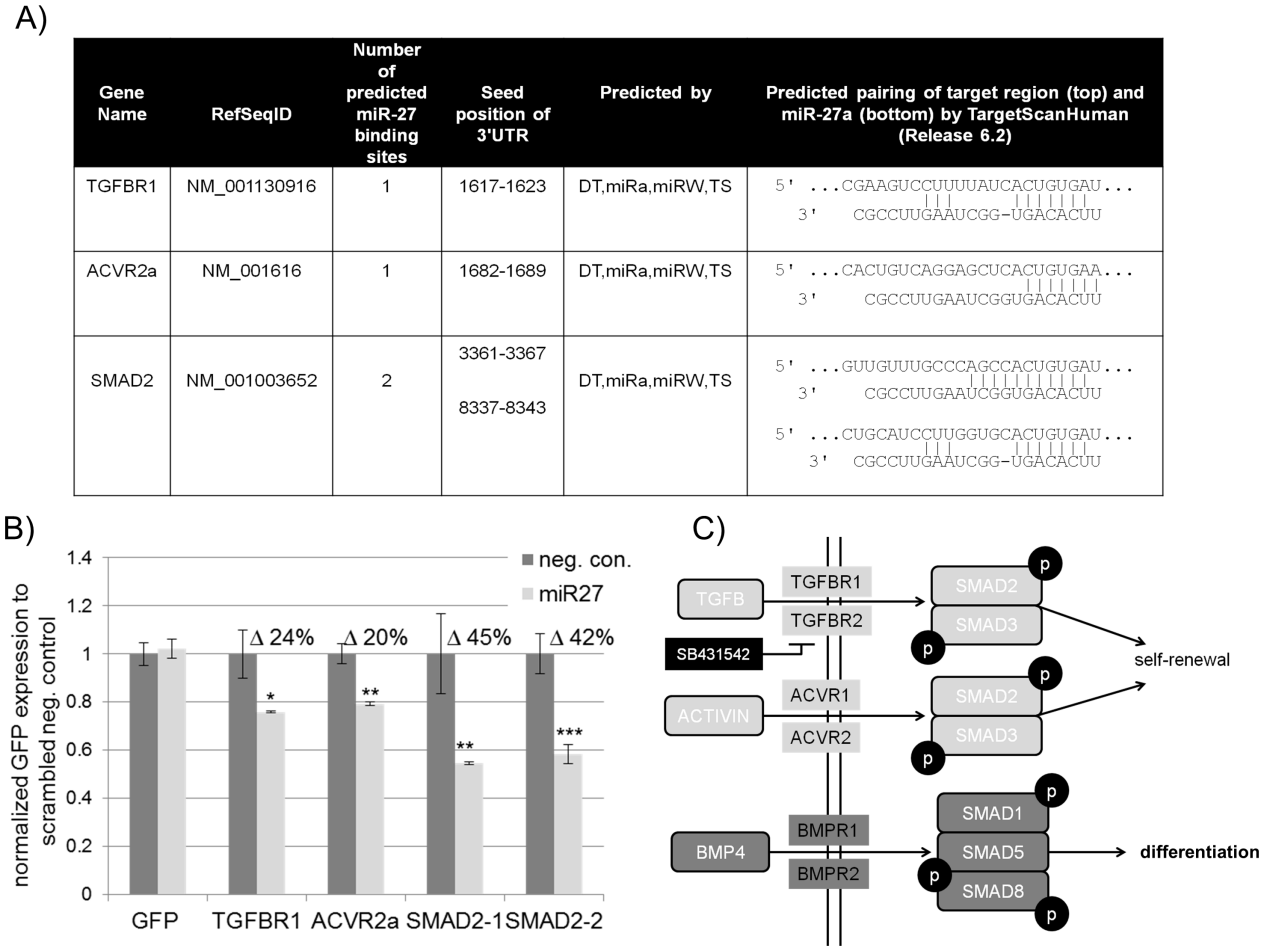
miR-27 directly inhibits a number of genes of the TGFß signalling pathway that promote self-renewal in undifferentiated embryonic stem cells. (A) Table shows putative miR-27 target genes associated with TGFß-signalling as predicted by Diana Micro-T (DT), MiRanda (miRa), MirWalk (miRW) and TargetScan. (B) Normalized GFP expression (48 hours post transfection) of HEK293 cells co-transfected with EGFP-sensors bearing parts of the 3′-UTR of *TGFßR1*, *ACVR2a*, *SMAD2* or the 3′-UTR of the empty eGFP-vector (lane 1)together with either miR-27 mimics or a scrambled negative control mimic (neg. con.). All transfections were performed twice in biological triplicates (n = 6). An unpaired two tailed t-test was performed to reveal significant differences (*p<0.05, ** p<0.01, *** p<0.001). (C) Schematic representation of the TGFß-signalling cascade adopted from the KEGGs pathway database (www.genome.jp/kegg/pathway.html).

TGFß signalling is crucial for maintaining self-renewal and pluripotency. TGFß, ACTIVIN and NODAL activate their own receptors TGFß-R, ACTIVIN-R and NODAL-R, which in turn phosphorylate SMAD2 and SMAD3. Phosphorylated SMAD2/3, together with OCT4 induce expression of pluripotency associated genes such as *NANOG*. First, we employed a bioinformatic approach to identify putative miRNAs that might inhibit the SMAD2/3 branch of TGFß signalling. By using miRNA target gene prediction tools like TargetScan (www.targetscan.org) or DianaT (www.microrna.gr/tarbase/), we found that miR-27 is predicted to regulate two genes, *ACVR2* and *TGFßR1* which act upstream of the SMAD2/3 signalling cascade. Moreover, we found two putative binding sites within the 3′-UTR of *SMAD2* (Figure 1A). In order to validate these three genes as *bona fide* miR-27 targets, we generated GFP-sensor constructs bearing parts of the 3′-UTR with the putative miR-27 binding site as previously described. [16] The fact that *SMAD2* has been predicted to contain two miR-27 binding sites located ∼5 kb apart to each other, we decided to clone two sub-fragments of the SMAD2-3-′UTR within the 3′-UTR of the GFP-sensor plasmid (SMAD2-1 and SMAD2-2) to assure that the GFP-SMAD sensor is not regulated by endogenously expressed miRNAs. As a confirmatory experiment, we transfected HEK293 cells with the GFP-sensor and pdsRED as a control to monitor transfection efficiency, together with miR-27 mimics (Ambion) or a scrambled negative control miRNA mimic. We chose HEK293 cells for the miRNA target gene sensor approach because of their high transfection efficiency. To exclude that miR-27 does not influence the GFP-sensor *per se,* we performed a co-transfection of the GFP-sensor with miR-27 or the scrambled negative control. In both cases we did not observe significant differences in GFP expression (Figure 1B). However, in the case of TGFßR1, we observed a significant reduction of 24% (p = 0.0138) in GFP expression in the presence of miR-27. Over-expression of miR-27 led to a ∼20% decrease in GFP expression of the GFP-ACVR2a sensor (p = 0.00096). The highest GFP repression was observed for both SMAD2 constructs in the presence of miR-27. We observed 45% repression of the first binding site (SMAD2-1) and a 40% repression in the case of the second (SMAD2-2). Taken together, we have been able to confirm that miR-27 targets the 3′-UTRs of *SMAD2* and their upstream activators, *TGFßR1* and *ACVR2A*. These results show that miR-27 might act as a negative regulator of pluripotency because all three genes are known regulators of self-renewal in human ES/EC cells, as illustrated in Figure 1C.

### miR-27 targets the pluripotency-associated genes *NR5A2, POLR3G*, *LIN28B* and *NANOG*

**Figure 2:**
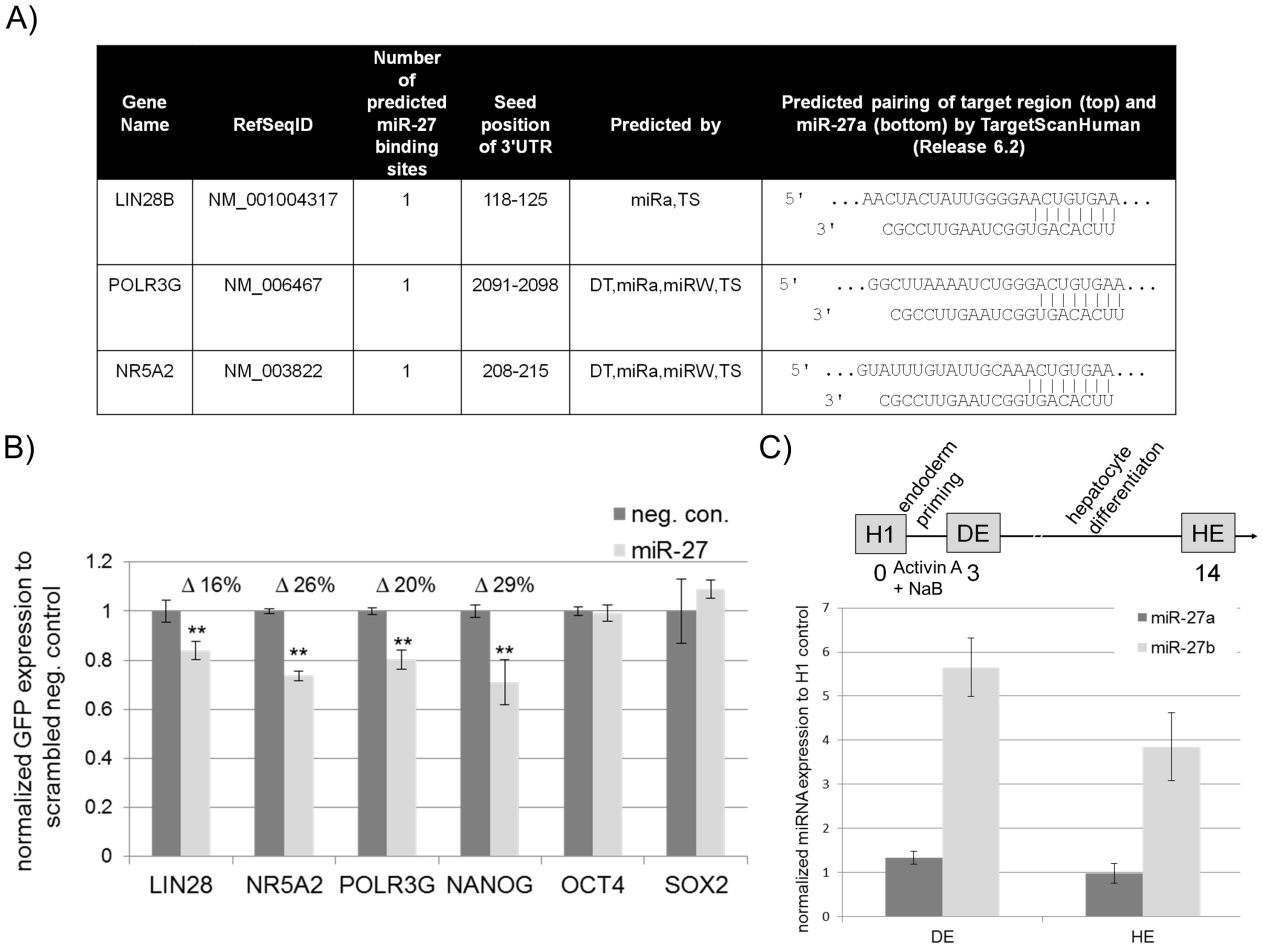
miR-27 directly inhibits a number of genes reported to sustain self-renewal in embryonic stem cells. (A) Table showing three putative miR-27 target genes predicted by Diana Micro-T (DT), MiRanda (miRa), MirWalk (miRW) and TargetScan that maintain self-renewal. (B) Normalized GFP expression (48 hours post transfection) of HEK293 cells co-transfected with EGFP-sensors bearing parts of the 3′-UTR of *LIN28B*, *NR5A2*, *POLR3G*, *NANOG, SOX2 or OCT4* together with either miR-27 mimics or a scrambled negative control mimic (neg. con.). All transfections were performed twice in biological triplicates (n = 6) and GFP expression measured by flow cytometry. An unpaired two tailed t-test was performed to reveal significant differences (** p<0.01). (C) Upper row: Schematic timeline of hESC (H1) undergoing hepatic differentiation. Total RNA was isolated at day zero (undifferentiated H1), three days after endoderm priming (DE) and 14 days after hepatocyte differentiation (HE). Lower row: miR-27 expression was carried out for miR-27a and miR-27b using TaqMan-based PCR on total RNA samples from the above described stages, DE and HE and normalized to the untreated/undifferentiated H1 control.

The fact that miR-27 regulates the SMAD2/3 branch prompted us to search for additional pluripotency-associated genes that might be regulated by miR-27. Screening with TargetScan we identified LIN28B as a putative miR-27 target gene (Figure 2A). Two isoforms of LIN28 have been described, the predominantly cytoplasmic expressed LIN28A and the nucleus-enriched LIN28B. Both isoforms have been reported to inhibit processing of miRNA let-7, a well-studied miRNA that promotes differentiation. Within the nucleus, LIN28A blocks the cleavage of the primary let-7 transcript by the microprocessor complex into the precursor forms, whilst LIN28B binds the precursor forms of let-7 and prevents let-7 maturation by the ribonuclease DICER [16], [17], [38]. By using the above described GFP-sensor assay, we observed a significant (16%) reduction in GFP expression (p = 0.008) in the presence of exogenous miR-27 compared to the negative control, thus suggesting that LIN28B is a direct target of miR-27 (Figure 2B).

Another candidate, the orphan nuclear receptor *NR5A2*, has been predicted to be a target of miR-27. NR5A2, also known as the liver receptor homolog LRH1, has been reported to be enriched in embryonic stem cells and promotes, co-operatively with SOX2, the expression of *OCT4* and *NANOG* [39]. Reprogramming studies revealed that ectopic expression of *Nr5a2* together with*Oct4*, *Sox2*, *Klf4*, and *Myc* enhances the reprogramming efficiency of mouse fibroblasts into induced pluripotent stem cells [9]. Moreover, a recent study reported that Oct4 can be substituted by Nr5a2 in inducing pluripotency in mouse fibroblasts [10]. Employing the GFP-sensor assay, we were able to confirm that miR-27 indeed directly regulates NR5A2 expression (Figure2B).

Another potential miR-27 target gene, the RNA polymerase III (Pol III) subunit *POLR3G*, a downstream target of OCT4 and NANOG, has been reported to promote the undifferentiated state of embryonic stem cells. It has been shown that loss of POLR3G promotes differentiation of hESC and iPS [11]. With the GFP-sensor assay, we observed a significantly reduced (∼20%) level of GFP expression from the GFP-POLR3G reporter induced by miR-27 over-expression (Figure 2B).

An interesting observation, when screening for miR-27 target sites, was that miR-27 sites are often predicted to be binding sites for miR-128 and *vice versa*. MicroRNAs have been reported to recognize their targets mainly through the seed region (nucleotides 2-7). Interestingly, the seed sequences of miR-128 (CACAGUG) and miR-27 (UCACAGU) overlap but are not identical. However, the seed sequence of miR-128 (CACAGUG, nucleotides 2–7) can also be found within the miR-27 sequence (U**CACAGUG**, nucleotides 3–8). *NANOG*, a downstream target of activated SMAD2/3, has been predicted to be a miR-128 target, but not a miR-27 target gene, by TargetScan. This observation inspired us to investigate if NANOG is indeed a target of miR-27. To confirm this, we generated a GFP-sensor construct bearing the 3′-UTR of NANOG and performed a co-transfection in HEK293 cells with either a scrambled negative control or miR-27. Surprisingly, miR-27 was able to repress GFP expression (approximately 29%) of the GFP-NANOG reporter compared with the negative control, thus indicating that NANOG is directly regulated by miR-27 (Figure 2B). Additionally, we generated two GFP-sensor constructs bearing the whole 3′-UTRs of SOX2 and OCT4, two genes not predicted to be miR-27 targets. For both constructs, we did not observe any significant changes in GFP-expression between miR-27 and the scrambled negative control (Figure 2B).

### miR-27 expression in hESC line H1 during hepatocyte differentiation

Since miR-27 directly inhibits a number of pluripotency-associated genes that are involved in silencing the SMAD2/3 branch of the TGFß signalling pathway, we postulated that miR-27 expression would be activated at an early time point during directed differentiation of pluripotent cells. In order to detect and quantify miR-27 expression, we performed RT-PCR on total RNA samples using TaqMan probes detecting miR-27a and miR-27b. A previous study reported that miR-27 is up-regulated in the hESC line CHA-4, undergoing hepatocyte differentiation [30]. We previously demonstrated the successful differentiation of anotherhESC line, H1, into hepatocyte-like cells [40]. First, we used total RNA samples from undifferentiated ES-cells at day zero (H1), differentiated cells three days after definitive endoderm (DE) and 14 days after hepatic endoderm (HE) induction (Figure 2C scheme). Thereafter, miRNA TaqMan assays revealed that miR-27a expression was just slightly activated in definitive endoderm cells (DE) while miR-27b expression was activated more than ∼5-fold compared to undifferentiated hESC (Figure 2C histogram). In hepatic endoderm cells (HE), 14 days after the initial differentiation, we observed no changes in miR-27a expression compared to the undifferentiated stage but a ∼4-fold increase of mature miR-27b. These results confirm that miR-27b expression is activated early during hepatic endoderm differentiation of embryonic stem cells.

### RNAi-mediated suppression of OCT4 in hESC induces miR-27a/b expression

**Figure 3:**
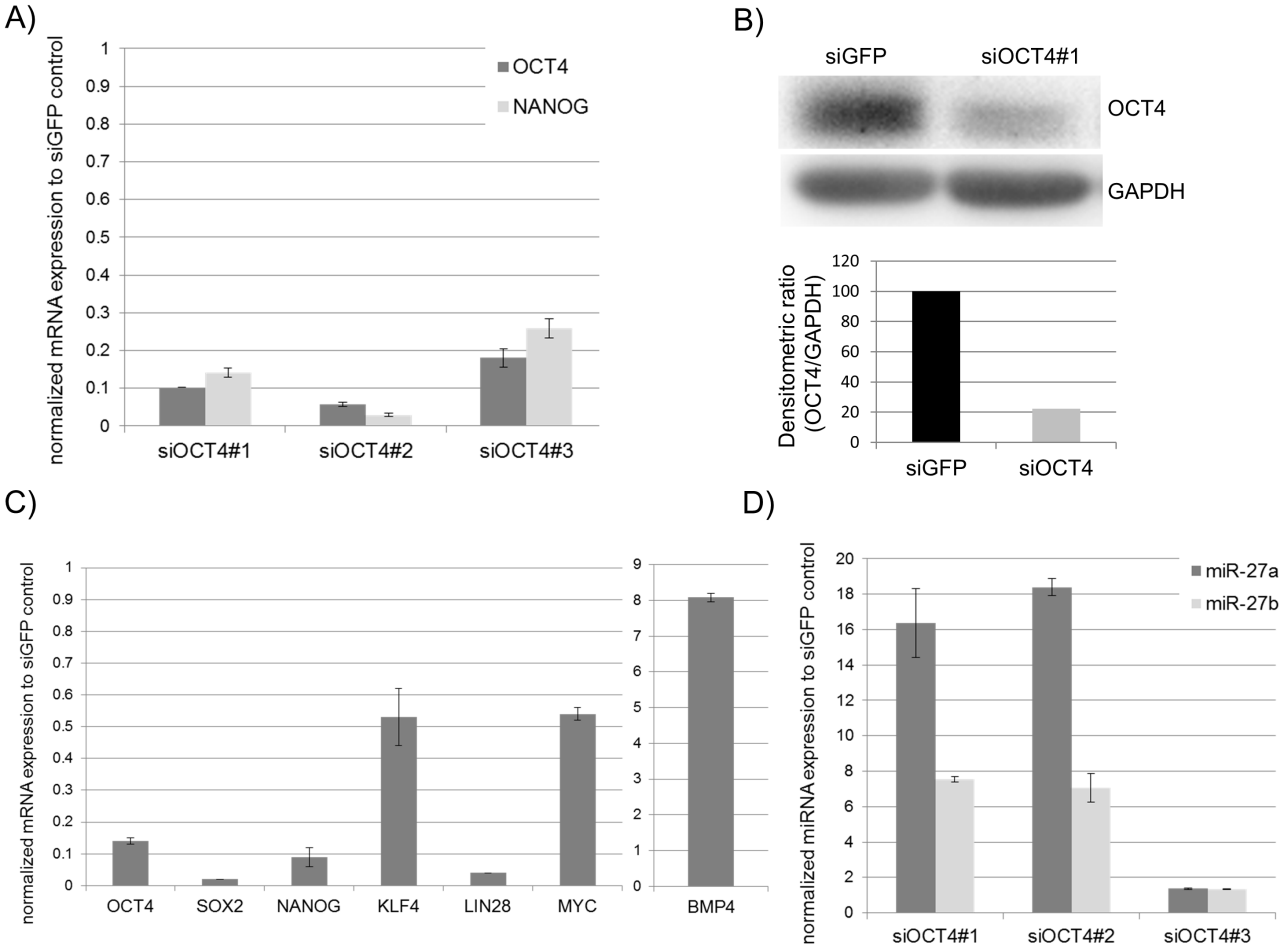
OCT4 knockdown in the hESC line H1 leads to activation of miR-27a and miR-27b expression. Successful OCT4 knockdown in hESC cells transfected twice with siRNA targeting either OCT4 or EGFP 72 h post transfection and confirmed by real-time PCR (A) and Western Blotting (B). (A) Relative *OCT4* and *NANOG* expression of three biological OCT4 knockdown samples (siOCT4#1-3) normalized to the siGFP knockdown control. (B) Western Blot analysis of OCT4 protein levels carried out for sample (siOCT4#1) and siGFP control sample with densitometric quantification (OCT4/GAPDH) (C) Relative expression of pluripotency-associated genes validated by real-time PCR for sample siOCT4#1 normalized to the siGFP knockdown control. (D) miR-27 expression was carried out using TaqMan-based PCR for all three biological siOCT4 samples and normalized to the siGFP control sample.

Ablating the function of OCT4 in hESC leads to reduced expression of pluripotency-associated genes such as *NANOG, LEFTY1, LEFTY2* and *NODAL*. A consequence of this is the up-regulation of the SMAD2/3 signalling antagonists, *BMP4* and *BMPR1* thus leading to the activation of the SMAD1/5/8 branch of the TGFß-signalling pathway and promoting the expression of somatic enriched genes [41]. To investigate miR-27 expression after RNAi-mediated knockdown of OCT4 inhESC, we isolated total RNA 72 h post transfection with siRNAs targeting either OCT4 (siOCT4) or EGFP (siEGFP) as a negative control [41]. To confirm the successful knockdown of *OCT4*, we quantified the expression of *OCT4*, and its downstream target *NANOG*, by RT-PCR in three biological replicates (Figure 3A). We observed a ∼80- to ∼95% repression of *OCT4* and ∼75 to ∼95% reduced *NANOG* expression in all three biological replicates (siOCT#1-3). Successful knockdown of OCT4 was also confirmed by western blotting for the representative sample (siOCT4#1) (Figure 3B). Densitometry analysis revealed an ∼80% reduction in the level of OCT4 protein 48 h post transfection (Figure 3B). Further RT-PCR analyses revealed decreased expression of the pluripotency-associated genes, *OCT4, SOX2, NANOG* and *LIN28* and an expected increased expression of *BMP4*, compared to the siEGFP control transfection (Figure 3C). Finally, we compared miR-27 expression in hESC 72 h after siRNA mediated knockdown of OCT4 with a TAQman miRNA assay. We observed for two samples with the most efficient OCT4 knockdown, a more than 16-fold increase in the levels of miR-27a and more than 6-fold increase in miR-27b expression (Figure 3D). The lowest, just 1-fold up-regulation of both, miR-27a and miR-27b, was observed with the less efficient OCT4 knockdown sample- siOCT4#3. These results confirm that OCT4 expression negatively correlates with miR-27a/b expression in hESC.

### miR-27 inhibits OCT4 and LIN28 expression at the transcriptional and translational level in embryonal carcinoma (EC) cells

In a next step, we wanted to investigate whether miR-27 over-expression promotes differentiation in hESC. Unfortunately, hESC cannot be efficiently transfected with either siRNAs or miRNAs by lipofection. An exception seems to be the previously presented lipofection of hESC with siRNAs targeting OCT4, resulting in a rapid loss of OCT4 both at the transcriptional and translational level 72 h post transfection (Figure 3). This domino effect can be explained by the fact that loss of OCT4, even at a moderate level, disrupts autocrine activation of important signalling pathways promoting self-renewal, such as FGF signalling, which in turn, results in decreased OCT4 expression of untransfected adjacent cells.

**Figure 4:**
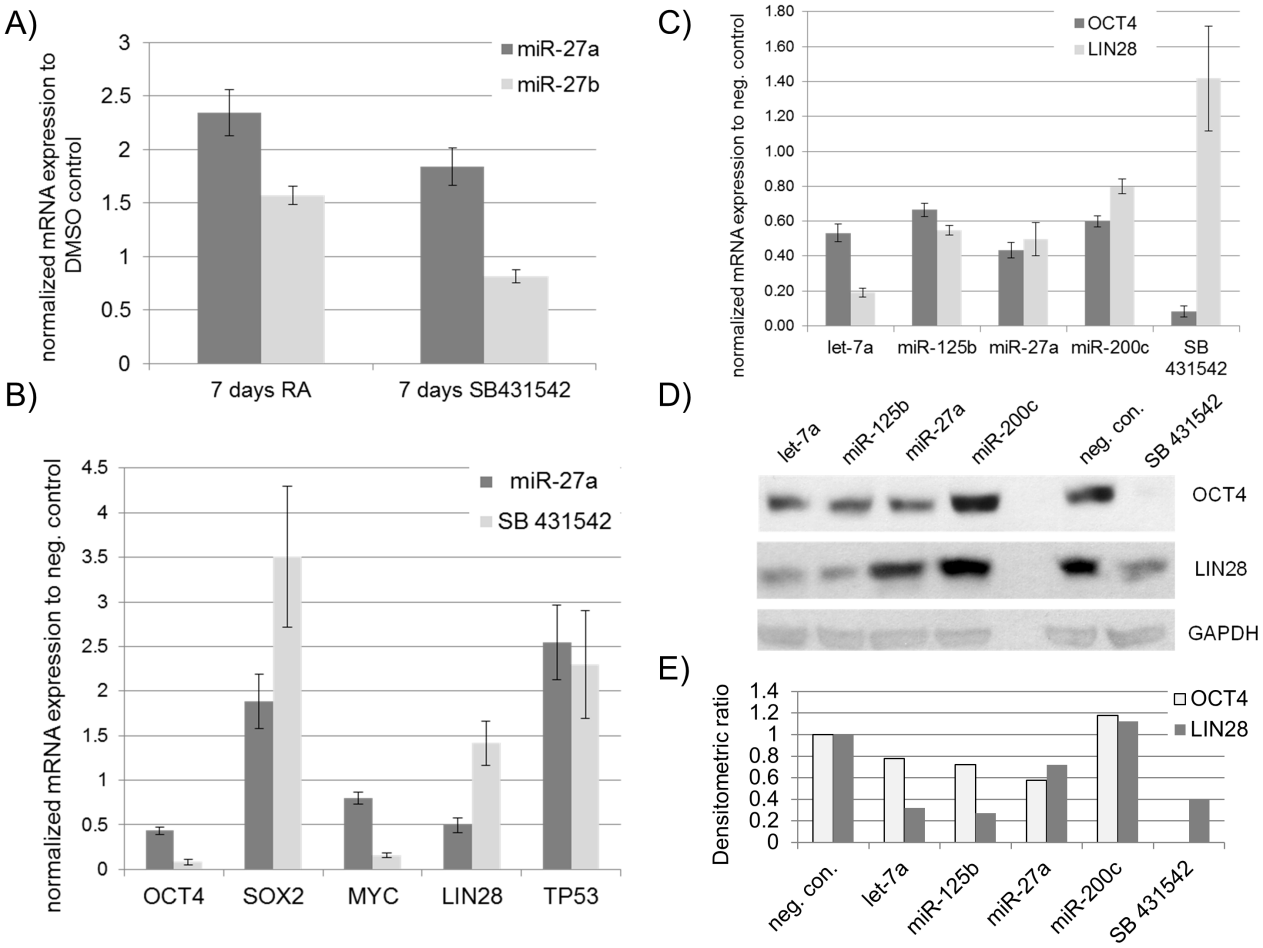
miR-27 inhibits OCT4 and LIN28 expression at both the transcriptional and translational level in embryonal carcinoma cells (NCCIT). (A) Analysis of miR-27 expression was carried out for miR-27a and miR-27b using TaqMan-based PCR on total RNA samples isolated from NCCIT cells undergoing RA stimulated neuronal differentiation for seven days or by blocking TGFßR2 with SB431542 for seven days and normalized to the DMSO-treated control. (B) qRT-PCR of selected genes (log2-fold change relative to the negative control) was validated for NCCIT cells transfected once with miR-27 or treated with SB431542 for 48 h. NCCIT cells transfected with a scrambled miRNA mimic was used for normalization. (C) Relative *OCT4* and *LIN28* expression in NCCIT cells transfected with scrambled negative control miRNA mimics, let-7a, miR-125b, miR-27a, miR-200c or treated with SB431542 for 72 h and validated by qRT-PCR. (D) Western Blot analysis of OCT4 and LIN28 expression in NCCIT cells treated as described in (C). (E) Normalized densitometric-derived ratios of Western Blot presented in (D).

Therefore, for miR-27 over-expression studies, we chose the human embryonal carcinoma cell line NCCIT. Human embryonal carcinoma cells (hEC), have substantial similarities with hESC with respect to self-renewal, gene expression signature (e.g. OCT4, LIN28 and NANOG), surface antigens as well as alkaline phosphatase activity [3]. Since induced differentiation towards definitve endoderm resulted in an increase in miR-27a expression in hESC, we performed two additional differentiation experiments using our hEC model. First, we treated NCCIT cells for one week with retinoic acid (RA), leading to differentiation towards the neuro-ectoderm lineage [42]. Second, we treated NCCIT cells for one week with SB431542, a small molecule that has been shown to block TGFßR2, resulting in inactivation of the SMAD2/3 signalling branch leading to an activation of mesodermal markers and loss of self-renewal [43]–[45]. Since both molecules were solubilized in DMSO we used DMSO treated cells as a control. Using the miRNA TaqMan assay, we observed more than 2-fold increased expression of miR-27a and a moderate increase of miR-27b in the retinoic acid treatment compared to DMSO-treated NCCIT cells. Loss of self-renewal and therefore differentiation of hEC with SB431542 treatment resulted in ∼1.8-fold induced expression of miR-27a and an even lower level of miR-27b expression (Figure 4A). Successful blocking of TGFßR2 in NCCIT cells by SB431542 was validated by quantitative RT-PCR analysis of *OCT4* and *MYC levels*. Both genes were previously reported to be downstream targets of the TGFß/SMAD2/3 signalling cascade [6], [46]. Indeed, blocking of TGFßR2 for three days with SB431542 led to a 90% reduction in expression of *OCT4* and ∼80% reduced *MYC* level whilst *SOX2* and *TP53* expression was induced in NCCIT cells (Figure 4B). Next, we examined the influence of miR-27 in hEC. miR-27 over-expression in NCCIT cells repressed *OCT4* and *LIN28B* expression levels to about 50% in comparison to the scrambled miRNA negative control but however no reduction of *MYC* expression. In contrast, *SOX2* was ∼3.5-fold upregulated and *TP53* expression increased ∼2.5-fold (Figure 4B). The observation that miR-27 over-expression leads to a reduction in *OCT4* and *LIN28B* expression, led us to investigate whether miR-27 inhibits LIN28B or OCT4 at the protein level. To achieve this, we transfected NCCIT cells with miR-27 and isolated total RNA and protein 48 h post transfection. As additional positive controls, we transfected NCCIT cells with let-7a or miR-125b, two miRNAs that have been shown to directly inhibit LIN28B and indirectly repress OCT4 [16], [17], [47]. In addition to the scrambled miRNA as negative control, we also used miR-200c, which has been reported to promote pluripotency and enhance reprogramming efficiency of somatic cells towards induced pluripotent stem cells [48], [49].

At the transcriptional level, in the case of let-7a, we observed a ∼80% reduction of *LIN28B* expression and ∼50% reduced expression of *OCT4* (Figure 4C). miR-125b over-expression resulted in a moderately decreased expression of *OCT4* and *LIN28* in comparison to let-7a. Similar results were observed with transfection of NCCIT cells with miR-27a (Figure 4C). Transfection of NCCIT cells with miR-200c led to a non-significant decrease in the expression of both genes. The highest repression of *OCT4* was observed after blocking TGFßR2 with SB431542, whereas LIN28 expression was slightly increased (Figure 4C).

At the translational level, OCT4 and LIN28 levels were not altered after transfection with the scrambled negative control mimic (Figure 4D). Treatment of NCCIT cells with SB431542 for 48 h resulted in a drastic decrease in OCT4 levels. OCT4 and LIN28B expression were slightly induced in response to miR-200c over-expression (Figure 4D+E). As expected, let-7a and miR-125b strongly repress their target LIN28B and moderately inhibit OCT4 (Figure 4D+E). For miR-27 over-expression, the expression of OCT4 was highly reduced to levels similar to miR-125b and let-7a. In the case of LIN28, we just observed a moderate reduction compared to the negative control (Figure 4D+E).

### miR-27 over-expression in hEC activates expression of developmental-associated genes and represses pluripotency-associated genes at the transcriptional level

**Figure 5:**
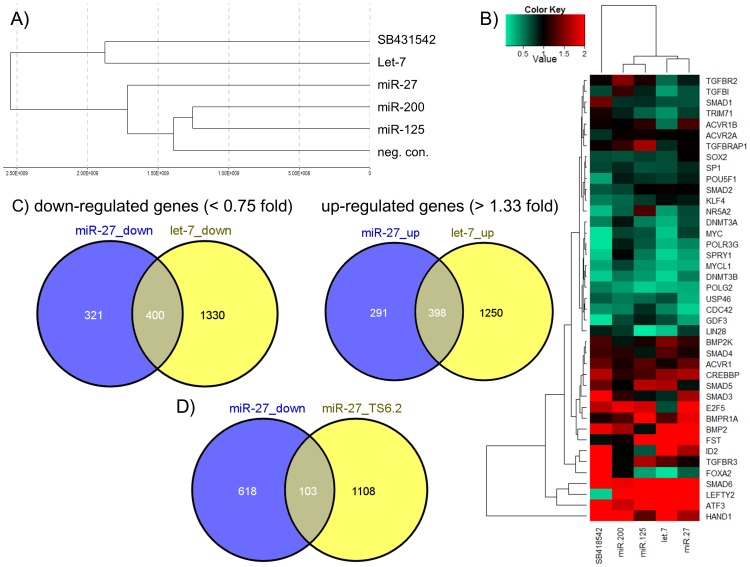
Transcriptome analysis of human embryonal carcinoma cells (NCCIT) post transfection with miR-27, let-7, miR-125 or miR-200. (A) Hierarchical clustering of NCCIT cells transfected either with miRNAs (miR-27, let-7, miR-125, miR-200 or neg. control mimic) or treated with the TGFßR2 inhibitor SB431542 (B) Heat map representing the expression of selected genes relative to the negative control transfection (Detection P-Value <0.01) (C) Venn diagrams representing the overlap of up- and down-regulated genes by let-7 and miR-27 (Detection P-Value <0.01) in comparison to the negative control transfection. (D) Venn diagram representing the overlap of down-regulated genes by miR-27 in comparison to miR-27 target genes predicted by TargetScan (human) V6.2.

In order to achieve a more global insight on the function of miR-27 during early development, we transfected hEC line NCCIT with miRNA mimics. By using the Illumina Beadstudio microarray platform, we analysed the transcriptomes of NCCIT cells transfected with the following miRNA mimics (let-7, miR-125, miR-27, miR-200 and a scrambled negative control) and also treated with the TGFßR2 inhibitor SB431542 after 72 h. The dendrogram in Figure 5A presents the correlation of the transcriptomes to each other. It shows that blocking TGFßR2 with SB431542 or the over-expression of let-7 has the strongest effect at the transcriptome level, compared to the negative control transfection, followed by miR-27 (Figure 5A). Smaller differences were observed for the miRNAs miR-125 and miR-200. The heat map illustrates transcriptional changes 72 h after over-expression of selected miRNAs (let-7, miR-125, miR-27, miR-200) compared to the scrambled negative control of a number of selected genes previously shown to promote either self-renewal (e.g. *LIN28, TRIM71, DNMT3A, DNMT3B*) or induction of differentiation (e.g. *SMAD6, BMP2, FST*). In the case of miR-27 over-expression, it is obvious that many pluripotency-associated genes were slightly or strongly down-regulated, whereas genes which promote differentiation were mainly up-regulated (Figure 5B). Similar tendencies were observed after let-7 over-expression, which led us to compare the overlap of up- and down-regulated genes between both miRNAs. We chose <0.75-fold and >1.33-fold as substantial thresholds in order to detect even slightly down- or up-regulated genes. As shown in Figure 5C, the Venn diagrams represent a high overlap of substantially up- and down-regulated genes induced or repressed by let-7 and miR-27 in hEC cells. 55% (400 of 721 genes) of all substantially <0.75-fold down-regulated genes after miR-27 over-expression were also down-regulated by the presence of let-7. Moreover, ∼58% (398 of 689 genes) of all substantially >1.33-fold up-regulated genes after miR-27 over-expression were up-regulated after ectopic expression of let-7. The complete list of miR-27 and let-7 regulated genes in NCCIT cells is presented in table S1. We also compared the number of genes down-regulated after miR-27 over-expression with miR-27 target genes predicted by Targetscan human V6.2 (Figure 5D). Here we revealed just a weak overlap of 14%.

Next, we analysed 721 substantially >1.33-fold up-regulated and 689 substantially <0.75 down-regulated genes (listed in table S1) after miR-27 over-expression with the online gene expression analysis tool “DAVID” in order to identify pathways regulated by miR-27. Therefore, we used a Pvalue <0.01 and Benjamini <0.05 as a threshold in order to identify significantly regulated pathways. The table reveals that miR-27 up-regulates a number of pathways associated with developmental processes, such as p53-, WNT- and TGFß-signalling (Table 1). Furthermore, miR-27 seems to act as a cell cycle regulator and mediator of cell-cell junctions. We also screened for a number of pathways down-regulated by miR-27 in NCCIT cells. Using again a Pvalue <0.01 and Benjamini <0.05 we were unable to identify significantly regulated pathways.

**Table 1:** List of pathways and associated genes significantly up-regulated 72 h after post-transfection of NCCIT with miR27.

Term	Count	%	PValue	Benjamini	Genes
hsa04350:TGF-beta signaling pathway	13	1.96	0.0001	0.0133	BMP2, E2F5, SMAD6, CREBBP, FST, RBL1, SMAD3, ID2, ID1, INHBE, ZFYVE9, LEFTY2, BMPR1A
hsa04310:Wnt signaling pathway	16	2.42	0.0006	0.0386	WNT5A, FZD8, TBL1XR1, PPP2R5A, CREBBP, PPP3R1, SMAD3, FZD3, DVL1, CTNNB1, CSNK2A2, CSNK2A1, CCND2, JUN, PLCB1, PLCB2
hsa04520:Adherens junction	11	1.66	0.0006	0.0274	CSNK2A2, FGFR1, TJP1, CSNK2A1, WASF3, FYN, CREBBP, SMAD3, WASL, SRC, CTNNB1
hsa05200:Pathways in cancer	26	3.93	0.0007	0.0212	WNT5A, FGFR1, E2F2, PML, FOXO1, PTEN, GLI3, CTNNB1, PTK2, BCL2, FZD8, BMP2, COL4A1, BCR, CREBBP, SKP2, SMAD3, FZD3, RB1, STAT3, DVL1, LAMA2, LAMA1, ITGA6, JUN, LAMC1
hsa05222:Small cell lung cancer	11	1.66	0.0013	0.0327	LAMA2, E2F2, LAMA1, PTK2, COL4A1, ITGA6, BCL2, SKP2, RB1, LAMC1, PTEN
hsa04510:Focal adhesion	18	2.72	0.0016	0.0353	COL4A1, TLN2, PPP1CB, PTEN, SRC, CTNNB1, LAMA2, LAMA1, PTK2, PDPK1, DOCK1, ITGA6, CCND2, FYN, JUN, BCL2, RAP1A, LAMC1

## Discussion

miR-27 has been recently reported to be involved in metabolic processes such as fatty acid metabolism, where miR-27 inhibits adipogenesis through targeting two core regulators of adipogenesis, the peroxisome proliferator-activated receptor gamma (PPARγ) and C/EBPalpha [33]. Additionally, miR-27expression has been linked to a number of diseases, such as neovascular age-related macular degeneration (AMD), where it has been reported to promote abnormal angiogenesis of the blood vessels behind the eye, by targeting the angiogenesis inhibitors SEMA6A and SPROUTY2 [50], [51]. miR-27 is involved in developmental processes. It promotes granulocyte differentiation through targeting the RUNX1 [35]. In mesenchymal stem cells (MSCs), miR-27 expression is increased and promotes osteoblast differentiation by inhibition of the adenomatous polyposis coli gene (*APC*), a known activator of the WNT signalling pathway [32]. Another study demonstrated that miR-27 is strongly up-regulated in the heart of neonate mice and promotes myocardic maturation through modulating Mef2c [52].

The findings of our study reveal a novel role for miR-27 as a negative regulator of self-renewal by inhibiting core factors associated with pluripotency in hEC cells. Our data has led us to hypothesise that miR-27 expression is activated upon the loss of self-renewal. The following evidences support our hypothesis. (i) We have shown that miR-27b expression increases up to 5-fold three days after endoderm priming of undifferentiated hESC line H1 (Figure 2C). This observation is consistent with a recent report where the authors demonstrate that miR-27b is up-regulated in another hESC line (CHA-4) after endoderm priming [30]. (ii) An up-regulation of expression of miR-23 in the human embryonal cell line (NT2) after RA treatment for 3 weeks [53]. As miR-23 is transcribed together with miR-24 and miR-27 in a polycistronic cluster, these results support our observation that expression of miR-27a increases in the hEC line (NCCIT) 7 days post RA treatment (Figure 4A). Our results (Figure 2C and 4A) also demonstrate that miR-27b is activated during endoderm differentiation. In contrast, miR-27a expression is more prominent during neuro-ectoderm differentiation.(iii) The most conclusive evidence that miR-27 might be a negative regulator of self-renewal pluripotency, is the observed activated expression of miR-27a (∼16-fold) and miR-27b (∼6-fold) 72 hours post siRNA mediated knockdown of OCT4 in the hESC line H1. This implies that OCT4 either directly or indirectly, negatively regulates miR-27 expression in hESC (Figure 3).

**Figure 6:**
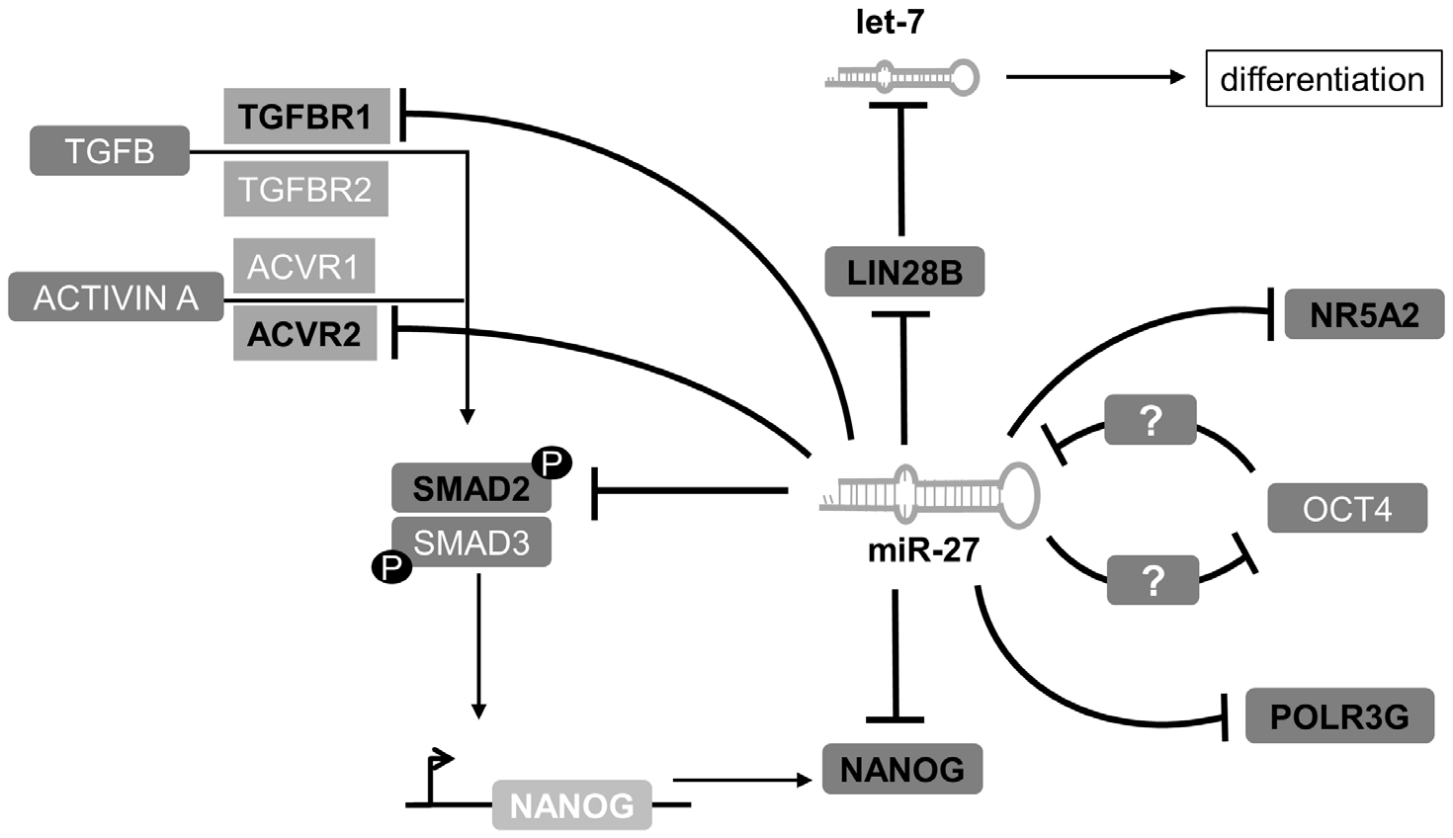
Schematic overview of our proposed regulatory network between miR-27 and pluripotency-associated genes. Genes highlighted in bold, black letters are those validated experimentally to be direct targets of miR-27.

We have confirmed with our GFP-sensor approach that miR-27 directly inhibits the ACTIVIN/NODAL branch of TGFß-signalling by targeting ACVR2A, TGFßR1 and SMAD2 (Figure 1C). These results reveal that miR-27 negatively regulates SMAD2/3 and therefore inhibits self-renewal in hESC. Interestingly, our group recently demonstrated that activated SMAD2/3 promotes the expression of pluripotency-associated genes such as *LEFTYA*, *LEFTYB*, *CER1* and *NODAL*. We also revealed that *NANOG* bears the SMAD2/3 binding motif within its promoter region [3]. Remarkably, we show here that miR-27 directly regulates NANOG by binding to its 3′-UTR and inhibiting its expression (Figure 2B). Additional evidence in support of miR-27 acting as an “off-switch” for self-renewal are as follows; (i) miR-27 moderately inhibits LIN28B by using the eGFP-sensor approach (Figure 2B). Additionally, over-expression of mir-27 in hEC cells represses LIN28 at the transcriptional and translational level (Figure 4C+D+E). LIN28B is a well-studied gatekeeper of pluripotency. LIN28B promotes OCT4 stability post-transcriptionally by interacting with polyribosomes in embryonic stem cells [14], [15]. Moreover, LIN28B binds the precursor form of miRNA let-7 and prevents its maturation [16], [17]. Let-7, one of the most potent differentiation-inducing miRNA, down-regulates a large number of pluripotency-associated genes such as, *LIN28*, *MYC*, *CDK1* and *HMGA2*at the translational level [16], [26], [27]. To summarize, our results imply that (i) miR-27 indirectly promotes Let-7 maturation by modulating LIN28B. (ii) miR-27 over-expression leads to an elevated expression of the tumor suppressor oncogene, *TP53* in hEC cells (Figure 4B). TP53 has been reported to activate miR-145, a repressor of pluripotency-associated genes such as *OCT4, SOX2, LIN28* and *KLF4* [28]. TP53 also promotes the expression of miR-34, another miRNA that has been reported to suppress reprogramming efficiency by inhibiting expression of SOX2 and NANOG [29]. (iii) Another tumor suppressor oncogene, *FOXO1*, has been reported to be regulated by miR-27. While over-expression of miR-27 results in a reduction of *FOXO1* expression in hEC-1B cells, inhibition of miR-27 with antagomiRs leads to a de-repression of *FOXO1* in Ishiwaka cells [36]. FOXO1 is an essential regulator of pluripotency. Loss of FOXO1 induces loss of expression of OCT4, NANOG, KLF4, SOX2, TRA-1-31 and TRA-1-60 in the hESC line H1 [54]. (iv) We have confirmed two transcription factors, POLR3G and NR5A2, as direct targets of miR-27 (Figure 2B). Previous reports demonstrated that NR5A2 binds co-operatively with SOX2 within the promoter regions of *OCT4* and *NANOG* and promotes their expression in embryonic stem cells [39]. In the case of the OCT4/NANOG downstream target *POLR3G*, it has been shown that down-regulation of POLR3G promotes differentiation of hESC [11]. (v) miR-27 has been reported to be linked to cancer progression where miR-27promotes cancer metastasis through epithelial–mesenchymal transition (EMT) in AGS cells [37], [55]. Moreover, miR-27 over-expression activates expression of ZEB1 and ZEB2, (two antagonist of E-CADHERIN), which then leads to activated ß-CATENIN expression and decreased E-CADHERIN levels [37]. E-CADHERIN is an important regulator promoting the undifferentiated state of hESC and is a key factor associated with the induction of pluripotency in somatic cells [56], [57]. Our transcriptome analysis revealed that over-expression of miR-27 in human embryonal carcinoma cells leads to down-regulation of pluripotency-associated genes, such as *GDF3*, *LIN28*, *TRIM71*, *DNMT3A*, *DNMT3B* and *USP46* and an activated expression of developmental genes such as *SMAD6*, *BMP2*, *FST* and *HAND1* (Figure 5C). We observed an increased expression of *LEFTY2*, a gene that has been previously reported to be abundantly expressed in hESC [58]. However, LEFTY2 is a key factor in various developmental processes and a previous knockdown study from our group reported an up-regulated expression of *LEFTY2* after siRNA mediated knockdown of OCT4 or NANOG in the embryonal carcinoma cell line NCCIT [3]. Moreover, it has been recently reported that the NODAL inhibitor, LEFTY2, is down-regulated by miR-302, a microRNA that is highly enriched in hESC, thus revealing that modulating LEFTY2 at the translational level might be important to promote the undifferentiated stage [59]. More strikingly, we observed an up-regulation of genes that control developmental pathways such as p53-, WNT- and TGFß-signalling after miR-27 over-expression in NCCIT cells (Table 1). Finally, we have shown that over-expression of miR-27 in hEC leads to a dramatic reduction in expression of OCT4 mRNA and protein (Figure 4C+D+E) but, as shown with the eGFP-sensor approach, OCT4 is not a direct target gene of miR-27 (Figure 2B). The fact that loss of OCT4 induces activation of miR-27 expression in hES and that miR-27 over-expression results in reduced OCT4 expression in hEC, might imply that OCT4 and miR-27 form an indirect negative feedback loop but OCT4 rather than miR-27, is required for the maintenance of self-renewal in pluripotent stem cells [41] as depicted in Figure 6.

### Conclusion

In summary, we have demonstrated

- Over expression of miR-27 in hEC leads to a down-regulation of OCT4 and LIN28 on the transcriptional and translational level.
- Loss of OCT4 expression and function in hES results in the induction of miR-27 expression.
- miR-27 directly targets a number of pluripotency-associated genes such as *TGFßR1, ACVR2, SMAD2, LIN28B, POLR3G*, *NR5A2 and NANOG*.

Therefore, we postulate that miR-27, a negatively regulated OCT4 target, is an inhibitor of self-renewal in hEC. However, both OCT4 and miR-27, operate in a larger differentiation mechanism that involves a negative feedback loop. Validation of this hypothesis is beyond the scope of this manuscript.

## Material and Methods

### Cell culture

HEK293 (ATCC CRL-1573) and NCCIT (ATCC CRL-2073) cells were cultured in high-glucose DMEM supplemented with 10% FCS and 2 mM glutamine. For differentiation assays, NCCIT cells were cultured in the presence of 10 µM retinoic acid (Sigma-Aldrich) or 2 µM SB431542 (Sigma-Aldrich) and the medium was changed daily over a period of one week. hESC line H1 were grown on BD Matrigel-coated plates in mouse embryonic fibroblast (MEF)-conditioned medium containing 8 ng/ml bFGF (Preprotech).

### Validation of miR-27 target genes

In order to search for miR-27 target genes, we used miRNA target gen prediction web tools such as TargetScan (http://www.targetscan.org/), miRanda (http://www.microrna.org), DIANA - microT-CDS (http://diana.cslab.ece.ntua.gr/micro-CDS/?r=search) and miRWalk (http://www.umm.uni-heidelberg.de/apps/zmf/mirwalk/). PCR fragments flanking the predicted miR-27 binding sites were cloned into the 3′-UTR of the modified EGFP-C1 vector. Primer sequences that have been used to generate EGFP-sensor constructs were designed using Primer3 program (http://frodo.wi.mit.edu/) and analysed in BLAST (http://blast.ncbi.nlm.nih.gov/Blast.cgi) for specificity (Table 2).

**Table 2:** Primers that have been used to generate GFP- miRNA target gene constructs. Restriction sites are highlighted in bold letters.

Primer	Sequence	Restriction site
TGFßR1fwd	atat**GCGGCCGC**CAGCTTTGCCTGAACTCTCC	*NotI*
TGFßR1rev	atat**CTCGAG**ATGGGGAAACACAGCTTATG	*XhoI*
ACVR2Afwd	atat**CTCGAG**ATTGATGGTAGGCAGGTGCT	*XhoI*
ACVR2Arev	atat**GAATTC**GAATGAGACCATGGGGACAC	*EcoRI*
SMAD2-1fwd	atat**CTCGAG**TTTATTGATGCCTGTTGTTTGC	*XhoI*
SMAD2-1rev	atat**GAATTC**GAATGAGACCATGGGGACAC	*EcoRI*
SMAD2-2fwd	atat**CTCGAG**ATGGCAAGTGAAGGAATTGG	*XhoI*
SMAD2-2rev	atat**GAATTC**TGACCAGAAATCACAGGACAT	*EcoRI*
LIN28Bfwd	atat**CTCGAG**GTTCTTTCCTTTACCCGGTTG	*XhoI*
LIN28Brev	atat**GAATTC**TCCAATTATATGAGAGAGGGTGTG	*EcoRI*
NR5A2fwd	atat**AAGCTT**TCTTCTAATTCAGATACTGGGGATTT	*HindIII*
NR5A2rev	tata**GAATTC**TTATGCTCTTTTGGCATGCAACATT	*EcoRI*
POLR3Gfwd	atat**CTCGAG**AACCACTTGAACCAGGCAAA	*HindIII*
POLR3Grev	atat**GAATTC** TTGGAACAAACATCCCTGCT	*EcoRI*
NANOGfwd	atat**CTCGAG**GGTCTTGGCTCACTGCAAG	*HindIII*
NANOGrev	atat**GAATTC**TTGGAACAAACATCCCTGCT	*EcoRI*
OCT4fwd	atat**GCGGCCGC**GTGCCTGCCCTTCTAGGAAT	*NotI*
OCT4rev	atat**CTCGAG**TCTACTGTGTCCCAGGCTTCT	*XhoI*
SOX2fwd	atat**GCGGCCGC**GGGGGAGAAATTTTCAAAGAA	*NotI*
SOX2rev	atat**CTCGAG**TCTCAAACTGTGCATAATGGAGT	*XhoI*

Validation of miR-27 target genes was performed as previously described [16]. Therefore, 5×10^4^ HEK293 cells were plated in monolayer per 12-well plate one day before transfection. Cells were transfected with 25ng pEGFP-sensor and 200ng pdsRed together with 10pmol miRNA mimic or negative control using Lipofectamine2000 reagent (Invitrogen). Cells were processed for flow cytometry (BD FACSCalibur) after 48 hours and further analysed with FLOWJO software (Tree Star Inc.). The geometric mean of GFP-positive cells was normalized to the scrambled negative control transfection. An unpaired two tailed t-test (n = 6) was performed to reveal significant differences (* p<0.05, ** p≤0.01, ***: p≤0.001).

### Transfection of hEC (NCCIT) with miRNA mimics

For miRNA over-expression studies in hEC, 4×10^5^ NCCIT cells were transfected with 6 µl Lipofectamine RNAiMax Reagent (Invitrogen) together with 50 pmol of the following Pre-miR miRNA precursors (# AM17100, Ambion): negative control #1, hsa-miR-200c-3p, hsa-let-7a-5p, hsa-miR-27a-3p or hsa-miR-125b-5p (all from Ambion).

### RNA Isolation

For TaqMan MicroRNA assays, total RNA was extracted using Trizol Reagent (Invitrogen) and further purified by phenol-chloroform extraction using Phase Lock Gel Heavy tubes (5PRIME). For qRT-PCR, total RNA was isolated using the MiniRNeasy Kit (Qiagen) and was digested with DNase I (Qiagen).

### miRNA TaqMan assay

20 ng of total RNA was reverse transcribed using the TaqMan MicroRNA Reverse Transcription Kit (Applied Biosystems) with specific RT-Primers for miR-27a (000408), miR-27b (000409) or RNU22 (001001). The reaction mixtures were incubated at 16°C for 30 min, 42°C for 30 min and 85° for 5 min. For quantitative PCR analysis the cDNA was diluted 1∶20 and 1.33 µl cDNA was mixed with 10 µl TaqMan 2× Universal PCR-Master Mix without Emperase UNG, 7.67 µl nuclease-free water and 1 µl 20× TaqMan MicroRNA assay for miR-27a (000408), miR-27b (000409) or RNU22 (001001) as an internal control. Four replicates were performed for each probe on an Applied Biosystems 7900HT Fast Real-Time PCR System using the following conditions: 95°C for 10 minutes and 40 cycles of 95°C for 15 sec followed by 60°C for 1 min. Data was further analysed with SDS software V2.4.1 (Applied Biosystems).

### Real Time-PCR analysis

RNA was reverse transcribed using M-MLV reverse transcriptase (Promega) and oligo(dT) primers. The cDNA was diluted 1∶20 and 2 µl were mixed with 5 µl of 2× SYBR Green PCR Mix (Applied Biosystems), 2 µl nuclease-free water and 250 nM of each primer (Table 3). Three replicates were performed for each probe and GAPDH was used as an internal control. Real-time PCR was performed on Applied Biosystems 7900 instrument using the following conditions: 95°C for 10 minutes and 40 cycles of 95°C for 15 sec followed by 60°C for 1 min. Data was further analysed with SDS software V2.4.1 (Applied Biosystems).

**Table 3:** Primers that have been used for quantitative Real-time PCR.

Gene name	Forward primer	Reverse primer
BMP4	TGAGTGCCATCTCCATGCTGTA	CGGCACCCACATCCCTCTACTA
GAPDH	CTGGTAAAGTGGATATTGTTGCCAT	TGGAATCATATTGGAACATGTAAACC
OCT4	GTGGAGGAAGCTGACAACAA	ATTCTCCAGGTTGCCTCTCA
NANOG	CCTGTGATTTGTGGGCCTG	GACAGTCTCCGTGTGAGGCAT
SOX2	GTATCAGGAGTTGTCAAGGCAGAG	TCCTAGTCTTAAAGAGGCAGCAAAC
MYC	CCAGCAGCGACTCTGAGGA	GAGCCTGCCTCTTTTCCACAG
TP53	CAGGGCAGCTACGGTTTCC	CAGTTGGCAAAACATCTTGTTGAG
LIN28B	AGCCCCTTGGATATTCCAGTC	AATGTGAATTCCACTGGTTCTCCT

### Microarray Transcriptome Analysis

Transfection of NCCIT cells with miRNA mimics and Total RNA extraction was performed as previously described above. The quality and concentration of the RNA samples was confirmed by Nanodrop and agarose gel analysis. For biotin-labeled cRNA production, 500 ng RNA was used with Illumina TotalPrep *RNA* Amplification Kit Expression BeadChip according to the manufactures instructions and hybridized onto an Illumina 8-Genechip V3. Single replicates were used for let7, miR-125, miR200 and SB431542. Duplicate samples were hybridized for miR-27 and the neg. control transfected NCCIT cells. After a series of washes the chip was stained with streptavidin-Cy3 and scanned using the Illumina Beadstation 500. Bead-summary data was generated using the Illumina Gene Studio Software Version 3. Bead-summary was processed via the R/Bioconductor environment employing packages lumi [60], limma [61], qvalue [62] and gplots. The quantile normalization algorithm was applied for normalization, limma p-value for determination of differential expression and qvalue for adjustment of the limma p-value for multiple testing. Genes that were substantially (DetectionPvalue <0.05/DiffLimmaQvalue <0.01) more than 1.3333-fold increased or less than 0.75-fold decreased after miR-27 over-expression compared to the negative control transfection were further screened for pathway related genes using the DAVID Bioinformatic Tool V6.7 [63].

### siRNA mediated knockdown in hESC line H1

H1 cells previously transfected with siRNAs targeting OCT4 or EGFP as a negative control were from our previously published OCT4 knockdown study [41].

### Differentiation of hESC line H1 towards hepatic endoderm

Total RNA samples from undifferentiated hESC at day zero (H1), three days after definitive endoderm (DE) and 14 days after hepatic endoderm (HE) were obtained using a modified protocol previously described by Hay et al. [64]. Therefore, hESC from (H1) were grown on BD Matrigel-coated plates in MEF-CM containing 8 ng/ml bFGF (Preprotech) until they reached 70% confluence. The media was exchanged to priming medium A (RPMI1640 (Invitrogen) containing 1× B27 (Invitrogen) 1 mM sodium butyrate (NaB) (Sigma-Aldrich) and 100 ng/ml activin A (PeproTech)). After 24 hours, the medium was exchanged again to priming medium A but with 0.5 mM NaB and cells were cultured for additional 48 hours. For definitive endoderm (DE) total RNA was extracted at this time point. For further hepatocyte induction the cells were cultured in hepatocyte differentiation medium A (DMEM (life technologies) supplemented with 20% Serum Replacement (life technologies), 1 mM glutamine (life technologies), 1% nonessential amino acids (life technologies), 0.1 mM β-mercaptoethanol (life technologies) and 1% DMSO (Sigma)) for five days. At day 8, the cells were cultured for additional six days in maturation medium B (L15 medium containing 8.3% fetal bovine serum (Gibco), 8.3% tryptose phosphate broth (Gibco), 10 µM Dexamethasone, 1 µM insulin (Sigma) and 2 mM glutamine (Gibco) containing 10 ng/ml hepatocyte growth factor (HGF) (Preprotech) and 20 ng/ml oncostatin M (OSM) (R&D systems).

### Western Blotting

Cells were lysed and sonicated in RIPA buffer, following SDS-PAGE of lysates. Proteins were transferred to Hybond nitrocellulose membranes (Amersham, GE Healthcare) and immunoblotted with anti-OCT4 antibody (1∶1,000; sc-5279; Santa Cruz Biotechnology), anti-LIN28B antibody (1∶1,000; #4196, Cell signalling) and anti-GAPDH (1∶10,000; #4300, Ambion). After washing with PBST, secondary HRP-linked antibody ECL Mouse IgG, (1∶5,000; Amersham) were used and detected with enhanced ECL reagent (Amersham). Figure 4D, Chemiluminescent Detection Films (Roche) and CURIX 60 film developing machine (Agfa) was used to visualize ECL-activity. Figure 3B, ECL-activity was detected with a FUSION-FX7 Advance Chemiluminescent system (Peqlab).

## Supporting Information

Table S1 **Microarray-based transcriptome analysis 72 h after miRNA over-expression in NCCIT cells.** Sheet A+B) Processed Data: The table represents statistical parameters for all Illumina probes. Bead-summary data have been generated with the Illumina BeadStudio for follow-up processing via the R/Bioconductor environment employing packages lumi, limma, qvalue and gplots. The quantile normalization algorithm was applied for normalization, limma p-value for determination of differential expression and qvalue for adjustment of the limma p-value for multiple testing. Differentially expressed genes where determined by the criteria: a) detection p-value <0.05 at least for treatment or control case, b) limma-q-value <0.05 and c) ratio <0.75 (for downregulation) or ratio >1.3333 (for upregulation). Genes significantly detectable (Detection Pvalue <0.01) are highlighted in red. Genes significant differentially expressed compared to the neg. control transfection (DiffPvalue <0.05), are highlighted in blue. The columns miR-X/neg. control represents the ratio between the AVG_Signal of miRNAs (let-7 and miR-27) compared to the AVG_Signal of the negative control. Genes more than 1.33-fold activated are highlighted in red, whereas genes less than 0.75-fold repressed are highlighted in green. Sheet C) sig. regulated genes The Table represents all significantly up- and down-regulated genes after miR-27 or let-7 over-expression in comparison to the neg. control transfection after 72 h in NCCIT cells with a Diff LimmaQval <0.05. The list of genes was used to screen for common up-and down-regulated genes presented in Figure 5c. D) DiffQ0.05common: The table provides a detailed list of common and unique >1.3333 fold up-regulated and <0.75 fold down-regulated genes (Official gene Symbol) by let-7 and miR-27 (Detection P-Value <0.05) of Figure 5C.
(XLSX)
